# YouTube as a Source of Patient and Trainee Education in Vascular Surgery: A Systematic Review

**DOI:** 10.1016/j.ejvsvf.2024.01.054

**Published:** 2024-01-26

**Authors:** Arshia Javidan, Muralie Vignarajah, Matthew W. Nelms, Fangwen Zhou, Yung Lee, Faysal Naji, Ahmed Kayssi

**Affiliations:** a Division of Vascular Surgery, Department of Surgery, University of Toronto, Toronto, Ontario, Canada; b Institute of Health Policy, Management, and Evaluation, University of Toronto, Toronto, Ontario, Canada; c Michael G. DeGroote School of Medicine, McMaster University, Hamilton, Ontario, Canada; d Temerty Faculty of Medicine, University of Toronto, Toronto, Canada; e Faculty of Health Sciences, McMaster University, Hamilton, Ontario, Canada; f Division of General Surgery, Department of Surgery, McMaster University, Hamilton, Ontario, Canada; g Harvard T.H. Chan School of Public Health, Harvard University, Boston, MA, USA; h Division of Vascular Surgery, Department of Surgery, McMaster University, Hamilton, Ontario, Canada

**Keywords:** Education, Systematic Review, Vascular Surgery, YouTube

## Abstract

**Objective:**

Due to its video based approach, YouTube has become a widely accessed educational resource for patients and trainees. This systematic review characterised and evaluated the peer reviewed literature investigating YouTube as a source of patient or trainee education in vascular surgery.

**Data sources:**

A comprehensive literature search was conducted using EMBASE, MEDLINE, and Ovid HealthStar from inception until 19 January 2023. All primary studies and conference abstracts evaluating YouTube as a source of vascular surgery education were included.

**Review methods:**

Video educational quality was analysed across several factors, including pathology, video audience, and length.

**Results:**

Overall, 24 studies were identified examining 3 221 videos with 123.1 hours of content and 37.1 million views. Studies primarily examined YouTube videos on diabetic foot care (7/24, 29%), peripheral arterial disease (3/24, 13%), carotid artery stenosis (3/24, 13%), varicose veins (3/24, 13%), and abdominal aortic aneurysm (2/24, 8%). Video educational quality was analysed using standardised assessment tools, author generated scoring systems, or global author reported assessment of quality. Six studies assessed videos for trainee education, while 18 studies evaluated videos for patient education. Among the 20 studies which reported on the overall quality of educational content, 10/20 studies deemed it poor, and 10/20 studies considered it fair, with 53% of studies noting poor educational quality for videos intended for patients and 40% of studies noting poor educational quality in videos intended for trainees. Poor quality videos had more views than fair quality videos (mean 27 348, 95% CI 15 154–39 543 views *vs.* 11 372, 95% CI 3 115–19 629 views, *p* = .030).

**Conclusion:**

The overall educational quality of YouTube videos for vascular surgery patient and trainee education is suboptimal. There is significant heterogeneity in the quality assessment tools used in their evaluation. A standardised approach to online education with a consistent quality assessment tool is required to better support online patient and trainee education in vascular surgery.

## Highlights

• YouTube videos for patient and trainee education in vascular surgery are in demand.
• Vascular surgery YouTube videos are generally poor to fair in educational quality.
• Videos worse in educational quality were viewed, on average, twice as frequently.
• The authors highlight key educational and research based areas for improvement.

## Introduction

The internet has become a widely accessed tool for entertainment, communication, and knowledge translation.^1^ With over two billion users and over 30,000 hours of uploaded video content per hour, YouTube (https://www.youtube.com/) is the second most visited website on the internet,^2^ and is widely accessible on multiple platforms (e.g., smartphones, tablets, computers, etc.). Although originally designed as an entertainment platform, YouTube has also become a highly accessed source of information for patients to learn about various disease processes and their management.^3^ Similarly, YouTube has become a commonly used educational tool for surgical trainees.^4^ Despite its popularity, the quality of medical educational material found on YouTube has been called into question. For disease processes in several surgical specialties, including neurosurgery,^5^ orthopaedic surgery,^6^ general surgery,^7^ and urology,^8^ YouTube videos have been shown to be of low educational quality for understanding procedural steps and correct disease management. For patient education, YouTube healthcare videos have often been found to contain misleading information across a breadth of pathologies.^3^

YouTube continues to grow in popularity owing to its accessibility, and sheer volume and breadth of publicly available videos.^9^ As such, patients and trainees alike will probably continue to access the platform for educational purposes. Patients may seek to educate themselves about their condition, prognosis, and expectations with surgical or non-surgical management.^10^^,^^11^ Similarly, trainees may use the platform to learn key procedural steps and how to expose procedure specific anatomy. While the educational quality of YouTube has been evaluated for several surgical and non-surgical specialties,^12^, ^13^, ^14^ a comprehensive evaluation has not yet been conducted in the field of vascular surgery. YouTube may play an especially important role for patient and trainee education in vascular surgery given the breadth of pathologies in the specialty, the often high complexity and comorbidity of disease burden, and the generally high technical requirements for trainees. Given that the platform will inevitably be accessed in high volumes, evaluation of this educational content, identification of areas of improvement, and a review of the literature is critically important. As such, the purpose of this systematic review was to characterise and evaluate the current literature investigating YouTube as a means of education for both patients and trainees as it relates to the field of vascular surgery.

## Materials and methods

### Study methodology and search strategy

This review was reported in accordance with the Preferred Reporting Items for Systematic Reviews and Meta-Analyses (PRISMA) statement.^15^ The search strategy was developed in consultation with a health sciences librarian and peer reviewed according to the Peer Review of Electronic Search Strategies (PRESS) guidelines.^16^ Comprehensive searches of the literature were conducted using the databases EMBASE, MEDLINE, and Ovid Healthstar from inception (1974, 1946, 1966, respectively) until 19 January 2023. The keywords, YouTube, health, healthcare, information, education, and surgery were used. Search strategies in their entirety can be found in Supplementary Table S1.

### Eligibility criteria

Studies were included if they were an original research article that evaluated or investigated YouTube as a source of patient or trainee education for any vascular surgery condition. Articles were excluded if they did not examine vascular surgery pathologies, did not use YouTube specifically as a video source, were narrative or systematic reviews (the citations of these papers were reviewed and extracted to the screening process), were commentaries, editorials, guidelines, news articles, or were written in a language other than English.

### Study screening and selection

All citations were imported into EndNote X7 (2016) and underwent de-duplication, which was manually confirmed. Study screening was performed in two stages. First, two reviewers (A.P.J., M.V.) independently screened the titles and abstracts of each article in accordance with the inclusion criteria. This was preceded by pilots of 100 studies each to ensure that inter-rater reliability (kappa) was greater than 0.6.^17^ Two reviewers (A.P.J., M.V.) then independently conducted full text screening. During both stages, discrepancies were resolved through joint discussion and consensus between the two reviewers and a senior author (F.N.).

### Data extraction and quality assessment

Data extraction occurred through standardised forms in Microsoft Excel in duplicate by two independent reviewers (A.P.J., M.V.). Discrepancies in data extraction were resolved through discussion with a senior author (F.N.).

### Study and video characteristics

Data extraction parameters included study meta-data (year of publication, publishing journal), methodological data (study objective, search criteria and search methodology used, type of analysis conducted), vascular surgery pathology, quantitative data regarding videos examined (video length, views), educational video quality, intended audience, and uploading source. Video uploader source was divided into the following categories: academic institution/affiliation, advertisers/commercial/media, healthcare organisation, patient/public, physician, other healthcare practitioner, other.

For studies that reported only median and interquartile ranges (IQR) or ranges, mean ± standard deviation (SD) were calculated based on methods proposed by Luo et al.,^18^ Shi et al.,^19^ and Wan et al.^20^ following normality tests. Studies with skewed quality scores were excluded from quantitative analysis. For studies with missing variance, analysis was completed using the largest reported SD.^21^

### Assessment of video educational quality

When quality of YouTube videos was assessed by a study, the overall educational quality of videos was categorised as either poor, fair, or good. Primarily this was determined by the authors’ global assessment in the study conclusion or discussion. If authors did not explicitly comment on the overall educational quality of the videos, the overall assessment was determined according to a validated assessment scale if one was used.

If a validated assessment scale was not used and an author generated scale was used instead, the quality was deemed to be poor if the total score was 33% or lower, fair if between 33% and 66%, and good if above 66%. The authors of this review did not watch or evaluate the quality of any of the individual videos included in the study set.

### Study quality appraisal

In contrast to educational quality of included videos in each study, quality appraisal of individual studies was conducted using the framework published by D'Souza et al. (M.V. and M.N.).^22^ This is a framework focusing on five overarching questions to assist authors in appraising studies using data from social media platforms. Each study was assessed according to the framework and a binary score (Y = Yes or N = No) was reported for each category. The overall study quality was graded as Good for a score ≥7 Fair, for a score ≤ six and ≥3, and Poor for a score ≤2.

### Statistical analysis

Quantitative pooling of means and proportions were conducted using the meta 5.2–0 package in R 4.1.2 (R Core Team, 2021, Vienna, Austria) with random effect, inverse variance weighting. Between study heterogeneity was determined using restricted maximum likelihood. Heterogeneity was quantified using *I*^2^ where 30% < *I*^2^ < 75% indicates moderate heterogeneity and *I*^2^ ≥ 75% indicates serious heterogeneity. Pooled means were expressed as raw mean and 95% confidence interval (CI), and pooled proportions were expressed as percentage and 95% CI. For studies that reported 0% or 100% proportion, a continuity correction factor of 0.5 was used to complete the analysis.

Descriptive statistics were used to evaluate trends and categorise data. Cohen's kappa was used to calculate inter-rater reliability. In all statistical tests, an alpha value of 0.05 was used to evaluate for statistical significance.

## Results

### Study selection

**Figure 1:**
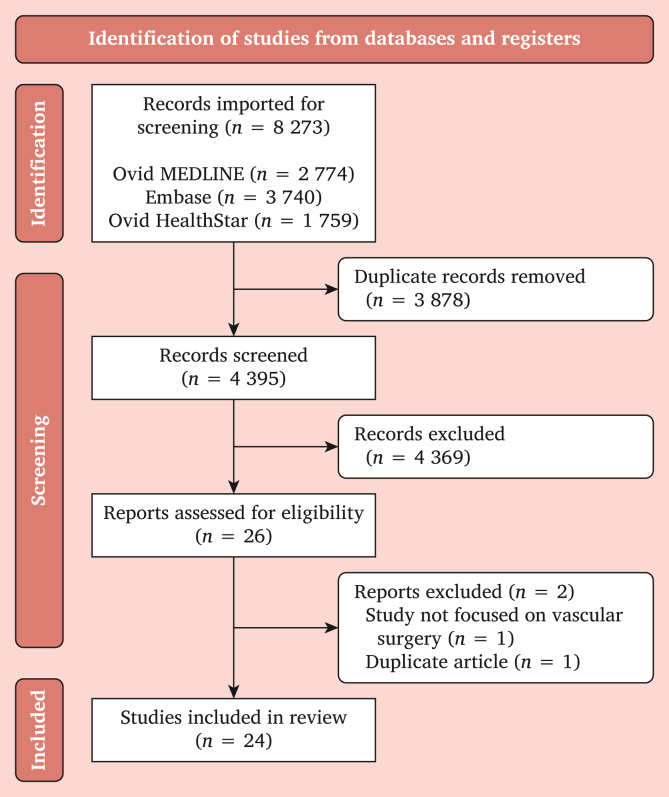
Preferred reporting items for systematic reviews and meta-analyses (PRISMA) flow chart.

The literature search across three databases yielded 8 273 initial citations (Fig. 1). Following the removal of 3 878 duplicate studies, a total of 4 395 citations remained for title and abstract screening. A total of 26 studies were evaluated in full text review, yielding a total of 24^23^, ^24^, ^25^, ^26^, ^27^, ^28^, ^29^, ^30^, ^31^, ^32^, ^33^, ^34^, ^35^, ^36^, ^37^, ^38^, ^39^, ^40^, ^41^, ^42^, ^43^, ^44^, ^45^, ^46^ studies for final analysis.

### Study characteristics

**Figure 2:**
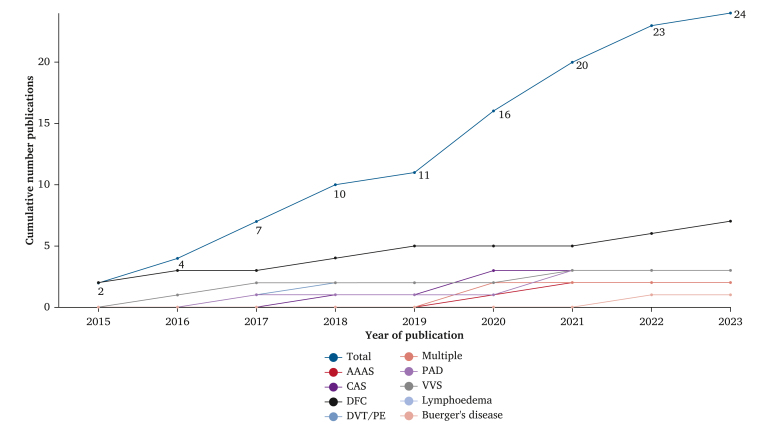
Cumulative publications by year of publication. AAA = abdominal aortic aneurysm; CAS = coronary artery stenting; DFC = diabetic foot care; DVT = deep vein thrombosis; PE = pulmonary embolism; PAD = peripheral artery disease; VV = varicose vein.

Articles were published between 2015 and 2023, with the majority of papers published in the last four years (Fig. 2). Individual study characteristics are described in Table 1. Overall, 15/24 (63%) studies had multiple raters for video selection. Of those that did, 12/15 (80%) calculated an inter-rater agreement and reported it. All 24 included studies performed a content analysis and none of them conducted a thematic analysis (Table 1).

**Table 1:** Summary of key findings from 24 included studies.

Study first author, year	Study title	Surgical pathology	Treatment(s) discussed	Targetted audience of videos	How was content analysed?	Multiple raters for video selection?	Inter-rater agreement reported?	Quality assessment of videos?	How was quality assessed?	Total videos	Video length in minutes	Views mean ± SD	Global assessment rating of quality
Dar, 2021^23^	An assessment of available information on the World Wide Web for patients with lower limb arterial disease	Peripheral arterial disease	Endovascular interventions, open surgical interventions	Patients	Content analysis, author created score and validated tools	Yes	Yes	Yes	JAMA, Global Quality Scale, author generated content assessment scale	22	6.2‡ ± 2.9‡	21 917.9‡ ± 29,800.6‡	Poor
Wu, 2021^24^	Investigation of the utility of web based videos about varicose veins	Varicose veins	Radiofrequency ablation, endovenous laser ablation, open surgery, sclerotherapy	Patients	Content analysis	Yes	No	No	No objective criteria reported, assessed for usefulness	205	NR	NR	NR
Günes, 2016^25^	YouTube as a source of information on varicose veins	Varicose veins	Open surgery, medical therapy, compression, sclerotherapy, endovenous laser ablation, laser, radiofrequency ablation	Patients	Content analysis, author generated criteria	Yes	Yes	Yes	Author generated usefulness scale	281	NR	NR	Fair
Radonjic, 2020^26^	YouTube as a source of patient information for abdominal aortic aneurysms	Abdominal aortic aneurysms	Endovascular aneurysm repair, open repair, surveillance	Patients	Content analysis, author generated score and validated tools	Yes	Yes	Yes	DISCERN, JAMA, author generated abdominal aortic aneurysm scale	51	7.6 ± 11.2	34 136 ± 79 134	Poor
Kwok, 2017^27^	YouTube as a source of patient information for varicose vein treatment options	Varicose veins	Endovenous laser ablation, high ligation and stripping, radiofrequency ablation, sclerotherapy, unspecified minimally invasive techniques	Patients	Content analysis, author generated score	Yes	Yes	Yes	Author generated scale using guidelines to assess information quality	228	NR	NR	Fair
Baytaroglu, 2021^28^	Characteristics of YouTube videos about peripheral artery disease during COVID-19 pandemic	Peripheral arterial disease	NR	Patients	Content analysis, validated tools	Yes	Yes	Yes	DISCERN, MICI	91	5.8‡ ± 3.7‡	40 973.9‡ ± 97 127.7‡	Poor
Yan, 2021^29^	Quality of procedural videos on endovascular repair of abdominal aortic aneurysm – viewer beware	Abdominal aortic aneurysm	Endovascular aneurysm repair	Trainees	Content analysis, author generated score	No	No	Yes	Author generated score on content, accountability, production	7	28.2∗ ± 53.7∗	57 986 (median)	Fair
Doenges, 2020^30^	Vascular surgery presence in carotid endarterectomy YouTube videos	Carotid artery stenosis	Carotid endarterectomy	Trainees	Content analysis, author generated score	No	No	Yes	Author generated score on content	46	9.7 ± 13.6	20 142 ± 50 426.6	Poor
Yammine, 2020^31^	Educational assessment of the major lower limb amputations videos on YouTube	Multiple	Major lower extremity amputations	Trainees	Content analysis, validated tool	Yes	Yes	Yes	Educational assessment tool	13	17.5§ ± 23.7‖	76 658.2§ ± 97,127.7‖	Fair
Yan, 2020^32^	Quality of carotid endarterectomy videos on YouTube: viewer be aware	Carotid artery stenosis	Carotid endarterectomy	Trainees	Content analysis, author generated score	No	No	Yes	Author generated score on content and production	23	NR	NR	Poor
Cousins, 2020^33^	Social media and online surgical education: a content analysis and descriptive study on quality of vascular surgery information available on YouTube	Multiple: Abdominal aortic aneurysms, carotid artery disease, peripheral arterial disease, lower limb amputation, varicose veins, deep vein thrombosis	NR	Patients	Content analysis, validated tools	No	No	Yes	DISCERN, HONcode, Global Quality Scale	600	NR	NR	NR
Smith, 2019^34^	Analysis of YouTube as a source of information for diabetic foot care	Diabetic foot	NR	Patients	Content analysis, author generated score	Yes	Yes	Yes	Author generated usefulness criteria	87	3.0§ ± 23.7‖	10 818.5§ ± 97,127.7‖	Fair
Park, 2018^35^	YouTube carotid endarterectomy videos: Are academic institutions keeping up with the content?	Carotid artery stenosis	Carotid endarterectomy	Trainees	Content analysis, author generated score	No	No	Yes	Author generated score on content	100	9.3‡ ± 13.5‡	16 229.7 ± 42 274.9	Fair
Kinariwala, 2018^36^	What is the public being told on YouTube about inferior vena cava filters?	Deep vein thrombosis/pulmonary embolism	Inferior vena cava filter placement (including open and endovascular removal)	Patients	Content analysis, author generated score	No	No	Yes	Author generated content score	99	2.2‡ ± 3.3‡	36 54.5‡ ± 18,684.1‡	Poor
De Araujo Nominato, 2018^37^	Analysis of Brazilian videos about diabetic neuropathy shared on YouTube	Diabetic foot	NR	Patients	Content analysis	Yes	No	No	NR	34	8.1§ ± 23.7‖	90 79.2§ ± 97,127.7‖	NR
Bademci, 2017^38^	YouTube as a potential source of information on deep venous thrombosis	Deep vein thrombosis/Pulmonary embolism	NR	Patients	Content analysis, author generated score	Yes	Yes	Yes	No objective criteria reported, assessed for usefulness	485	NR	18 790.6‡ ± 97,127.7‖	Fair
Pitcher, 2017^39^	Common femoral artery access on YouTube: What practices are being shown and who is delivering the message?	Peripheral arterial disease	Common femoral artery access	Trainees	Content analysis	No	No	No	NR	33	9.2‡ ± 23.7‡	2701.1§‡ ± 97,127.7‖	NR
Gupta, 2016^40^	Analysis of YouTube as a source of information for peripheral neuropathy	Diabetic foot	NR	Patients	Content analysis, author generated score	Yes	No	Yes	Author generated score on content	200	3.1†‡ ± 2.2†‡	1953.7†‡ ± 4,213.9†‡	Fair
Abedin, 2015^41^	YouTube as a source of useful information on diabetes foot care	Diabetic foot	NR	Patients	Content analysis, author generated score	Yes	Yes	Yes	Author generated usefulness criteria	89	3.3‡ ± 3.7‡	6,892.6‡ ± 23,520.1‡	Fair
Gupta, 2015^42^	YouTube and peripheral neuropathy: What patients want?	Diabetic foot	NR	Patients	Content analysis, author generated score	No	No	Yes	Author generated content criteria	200	NR	1,953.8†‡ ± 4,213.6†‡	Poor
Kücukakkaş, 2022^43^	Can YouTube be used as an educational tool in lymphedema rehabilitation?	Lymphoedema	Rehabilitation	Patients	Content analysis	Yes	Yes	Yes	Global Quality Scale, modified DISCERN	90	8.9 ± 10.5	35,165.6 ± 65 553.8	Poor
Doğan, 2022^44^	Diabetic foot care training and the presence of nurses in Turkish YouTube videos	Diabetic foot	NR	Patients	Content analysis, author generated checklist	Yes	Yes	Yes	Global Quality Scale, modified DISCERN, Author generated content criteria	87	8.1 ± 13.3	213.2 ± 366.6	Fair
Almaqhawi, 2023^45^	Evaluation of quality of diabetic foot examination on YouTube	Diabetic foot	NR	Patients	Content analysis	No	No	Yes	Global Quality Scale, JAMA, DUK-C	100	6.8 ± 6.6	101 311.9 ± 348 383.6	Poor
Çelik, 2022^46^	The reliability and quality of YouTube videos as a source of information on Buerger’s disease: a cross sectional study	Buerger's disease	NR	Patients	Content analysis	Yes	Yes	Yes	JAMA, Global Quality Scale, modified DISCERN	50	7.8 ± 10.1	41,071.6 ± 162 826	Poor

Data are presented as *n* or mean ± SD. SD = standard deviation; NR = not reported.

∗ Imputed from non-parametric data and not significantly skewed.

† Imputed from non-parametric data and significantly skewed.

‡ Combined mean ± SD from more than one group.

§ Mean value calculated by total value divided by the number of videos, or total value calculated by multiplying mean value and number of videos.

‖ Borrowed from the study that reported the largest SD.

Overall, most studies evaluated diabetic foot care (7/24, 29%), peripheral arterial disease (3/24, 13%), carotid artery stenosis (3/24, 13%), varicose veins (3/24, 13%), and abdominal aortic aneurysms (2/24, 8%)

### Video characteristics

Cumulatively, across all 24 papers, 3 221 videos provided a total of 123.1 hours of content and generated 37.1 million views. On average, studies analysed a mean of 134 videos. Overall, the pooled mean video length across all videos was 6.6 minutes (95% CI 5.8–8.0) with a pooled mean view count of 18867 (95% CI 11 233.1–26 500) (Table 2).

**Table 2:** Video characteristics based on topic discussed.

Disease state	Number of studies	Total videos	Total content – h	Pooled mean video length – min∗	Total views – millions	Pooled mean video views∗	Studies rated as poor quality/studies that performed quality assessment of videos	Pooled mean DISCERN/JAMA/GQS scores∗
Abdominal aortic aneurysms	2	58	9.8 *n* studies = 2 *n* = 58	8.0 (2.5–13.5) *n* studies = 2 *n* = 58 *I*^2^ = 3%	1.7 *n* studies = 1 *n* = 51	34136.0 (12 417.7–55 854.3) *n* studies = 1 *n* = 51 *I*^2^ = N/A	1/2 (50)	JAMA: 1.7 (1.5–1.9) *n* studies = 1 *n* = 51 *I*^2^ = N/A Modified DISCERN (/5): 2.4 (2.1–2.7) *n* studies = 1 *n* = 51 *I*^2^ = N/A GQS: N/A
Buerger’s disease	1	50	6.5 *n* studies = 1 *n* = 50	7.8 (4.9–10.6) *n* studies = 1 *n* = 50 *I*^2^ = N/A	2.1 *n* studies = 1 *n* = 50	41 071.6 (0.0–86 203.8) *n* studies = 1 *n* = 50 *I*^2^ = N/A	1/1 (100%)	JAMA: 2.1 (1.8–2.3) *n* studies = 1 *n* = 50 *I*^2^ = N/A Modified DISCERN (/5): 2.4 (2.0–2.7) *n* studies = 1 *n* = 50 *I*^2^ = N/A GQS: 2.6 (2.3–2.9) *n* studies = 1 *n* = 50 *I*^2^ = N/A
Carotid artery stenosis	3	169	22.9 *n* studies = 2 *n* = 146	9.4 (7.2–11.6) *n* studies = 2 *n* = 146 *I*^2^ = 0%	2.5 *n* studies = 2 *n* = 146	17 185.5 (9 982.7–24388.3) *n* studies = 2 *n* = 146 *I*^2^ = 0%	2/3 (67%)	JAMA: N/A Modified DISCERN (/5): N/A GQS: N/A
Deep vein thrombosis/pulmonary embolism	2	584	3.6 *n* studies = 1 *n* = 99	2.2 (1.5–2.8) *n* studies = 1 *n* = 99 *I*^2^ = N/A	9.5 *n* studies = 2 *n* = 584	10 696.5 (0.0–25 493.7) *n* studies = 2 *n* = 584 *I*^2^ = 90%	1/2 (50%)	JAMA: N/A Modified DISCERN (/5): N/A GQS: N/A
Diabetic foot care	7	797	47.2 *n* studies = 6 *n* = 597	5.6 (3.3–7.9) *n* studies = 5 *n* = 397 *I*^2^ = 86%	12.8 *n* studies = 7 *n* = 797	4 924.4 (0.0–11 493.4) *n* studies = 5 *n* = 397 *I*^2^ = 76%	2/6 (33%)	JAMA: 1.1 (0.9–1.4) *n* studies = 1 *n* = 100 *I*^2^ = N/A Modified DISCERN (/5): 2.7 (2.5–2.8) *n* studies = 1 *n* = 87 *I*^2^ = N/A GQS: 2.6 (2.1–3.0) *n* studies = 2 *n* = 187 *I*^2^ = 89
Lymphoedema	1	90	13.4 *n* studies = 1 *n* = 90	8.9 (6.7–11.1) *n* studies = 1 *n* = 90 *I*^2^ = N/A	3.2 *n* studies = 1 *n* = 90	35 165.6 (21 622.3–48 708.9) *n* studies = 1 *n* = 90 *I*^2^ = N/A	1/1 (100%)	JAMA: N/A Modified DISCERN (/5): 2.2 (2.0–2.4) *n* studies = 1 *n* = 90 *I*^2^ = N/A GQS: 2.7 (2.5–2.9) *n* studies = 1 *n* = 90 *I*^2^ = N/A
Peripheral arterial disease	3	146	16.1 *n* studies = 3 *n* = 146	5.9 (5.3–6.6) *n* studies = 3 *n* = 146 *I*^2^ = 0%	4.3 *n* studies = 3 *n* = 146	24 728.2 (7 200.1–42 256.4) *n* studies = 3 *n* = 146 *I*^2^ = 55%	2/2 (100%)	JAMA: N/A Modified DISCERN (/5): 2.2 (2.0–2.4) *n* studies = 1 *n* = 91 *I*^2^ = N/A GQS: N/A
Varicose veins	3	714	NR	NR	NR	NR	0/2 (0%)	JAMA: N/A Modified DISCERN (/5): N/A GQS: N/A
Multiple	2	613	3.8 *n* studies = 1 *n* = 13	17.5 (4.6–30.4) *n* studies = 1 *n* = 13 *I*^2^ = N/A	1.0 *n* studies = 1 *n* = 13	76 658.1 (23 859.9–129 456.4) *n* studies = 1 *n* = 13 *I*^2^ = N/A	0/1 (0%)	JAMA: N/A Modified DISCERN (/5): N/A GQS: N/A
Total	24	3221	123.1 *n* studies = 17 *n* = 1 199	6.6 (5.8–8.0) *n* studies = 16 *n* = 999 *I*^2^ = 90% *p* < .010†	37.1 *n* studies = 18 *n* = 1877	18 867.0 (11 233.1–26 500.8) *n* studies = 16 *n* = 1477 *I*^2^ = 89 *p* < .010^†^	10/20 (50%) *p* = .62‡	JAMA: 1.6 (1.1–2.2) *n* studies = 3 *n* = 201 *I*^2^ = 94% *p* < .010† Modified DISCERN (/5): 2.4 (2.2–2.6) *n* studies = 5 *n* = 369 *I*^2^ = 78% *p* < .010† GQS: 2.6 (2.4–2.8) *n* studies = 4 *n* = 327 *I*^2^ = 69% *p* = .79†

∗ Non-parametric data significantly skewed from normal were excluded. GQS = Global Quality Scale; N/A = not available.

† Subgroup interaction.

‡ Fisher's exact test.

Subgroup interactions between pooled mean video lengths of different vascular pathologies demonstrated statistically significant differences, driven largely by shorter videos for peripheral arterial disease (mean 5.9 minutes), diabetic foot care (mean 5.6 minutes), and deep vein thrombosis and or pulmonary embolism (mean 2.2 minutes). Subgroup interaction for pooled mean video views also showed statistically significant differences, driven by more views for videos related to peripheral arterial disease (mean 24,728), lymphoedema (mean 35 165), abdominal aortic aneurysms (mean 34,136), and multiple vascular pathologies (mean 76,658) (Table 2).

Overall, 41.3% of videos were uploaded by sources that would be considered educationally reputable (academic institutions, healthcare organisations, physicians, or other healthcare practitioner). Across all videos in the data set, 32.1% did not have a reported or recorded uploader source (Supplementary Table S4). Among videos uploaded specifically for trainee education, 53.5% did not report an uploader source (Supplementary Table S3). When sources were described, videos were most often uploaded by healthcare practitioners (15.1%), physicians (14%), advertisers, or commercial media (10%) (Supplementary Table S4).

### Assessment of video educational quality

Among all included studies, 21 separate quality assessment tools were used to evaluate video educational quality (Supplementary Table S2). Among the 20 studies which reported on the overall quality of educational content, 10/20 studies concluded it was poor, 10/20 studies indicated that it was fair, and 0/20 indicated that it was good (Table 1). Videos characterised as poor were on average viewed more often than videos characterised as fair in quality (mean 27 348 views *vs.* 11 372 views, *p* = .030, Table 3). In addition, videos intended for patients (mean 5.9 minutes) were generally far shorter than videos intended for trainees (mean 9.7 minutes) (Supplementary Table S3). Additionally, there was no difference between the proportion of videos that were poor in quality intended for patient education (8/15, 53%) compared with those intended for trainee education (2/5, 40%).

**Table 3:** Study characteristics based on video quality.

Global assessment of quality	Poor	Fair	Good	*p* value, between group difference‡
Studies	10	10	0	–
Videos	772	1577	0	–
Pooled mean video length	6.5 (4.9–8.2) *n* studies = 8 *n* = 549 *I*^2^ = 94%	6.8 (3.4–10.1) *n* studies = 10 *n* = 383 *I*^2^ = 85%	–	.90∗
Pooled mean views	27348.4 (15153.8–39543.0) *n* studies = 8 *n* = 549 *I*^2^ = 87%	11372.1 (3115.0–19629.3) *n* studies = 6 *n* = 861 *I*^2^ = 90%	–	.030∗
*Quantitative quality scores*				
JAMA	1.6 (1.1–2.2) *n* studies = 3 *n* = 201 *I*^2^ = 94%	–	–	–
Modified DISCERN	2.3 (2.1–2.4) *n* studies = 4 *n* = 282 *I*^2^ = 0%	2.7 (2.5–2.8) *n* studies = 1 *n* = 87 *I*^2^ = N/A	–	<.010∗
GQS	2.5 (2.3–2.8) *n* studies = 3 *n* = 240 *I*^2^ = 64%	2.8 (2.6–3.0) *n* studies = 1 *n* = 87 *I*^2^ = N/A	–	.11∗
*Source of upload*				
Academic affiliation/institution	2.0 (0.0–4.7) *I*^2^ = 69%	7.1 (0.0–14.1) *I*^2^ = 95%	–	.18∗
Advertisers/commercial/media	3.7 (0.0–8.0) *I*^2^ = 86%	18.0 (0.7–35.2) *I*^2^ = 99%	–	.12∗
Clinic/hospital/healthcare organisation	8.7 (0.0–23.4) *I*^2^ = 97%	4.5 (0.0–10.0) *I*^2^ = 94%	–	.60∗
Patient/public	4.1 (0.0–9.3) *I*^2^ = 88%	7.0 (1.2–12.7) *I*^2^ = 99%	–	.47∗
Physician	26.0 (6.8–45.1) *I*^2^ = 98%	2.5 (0.0–7.3) *I*^2^ = 94%	–	.020∗
Other healthcare provider	11.8 (1.3–22.4) *I*^2^ = 94%	18.0 (3.6–32.3) *I*^2^ = 98%	–	.50∗
Other	5.3 (0.0–11.2) *I*^2^ = 84%	15.1 (0.3–29.9) *I*^2^ = 98%	–	.23∗
Not reported	35.2 (5.7–64.6) *I*^2^ = 100%	27.8 (0.0–56.0) *I*^2^ = 100%	–	.72∗

Data are presented as *n*, *n* (%), 95% confidence interval. GQS = Global Quality Scale; N/A = not available.

∗ Subgroup interaction.

‡ Fisher's exact test.

### Study quality appraisal

When the quality of each included study was evaluated, 8/24 (33%) studies were found to be of good quality, with 16/24 studies (67%) being of fair quality (Supplementary Table S5).

## Discussion

### Key findings

This systematic review characterised YouTube as a source of patient and trainee education in vascular surgery. The review found that (1) videos were poor to fair in educational quality, with the studies themselves being generally of fair to good methodological quality; (2) videos characterised as being poor in educational quality are viewed on average more often than higher quality videos; (3) there has been an exponential increase in the volume of literature evaluating YouTube in vascular surgery; (4) there is substantial heterogeneity in the quality assessment instruments used in the literature; (5) apart from videos without an identified uploader, most videos were uploaded by physicians or other healthcare practitioners. Taken together, these findings suggest that YouTube is seeing increasing use for both patient and trainee education in vascular surgery, but the overall educational and methodological quality of such information is lacking.

As evidenced by the 37.1 million views in this study, educational vascular surgery videos are in demand by both patients and trainees. Despite the popularity of YouTube, the overall educational quality of the videos is generally of poor to fair. It is also somewhat concerning that videos characterised as being poor in educational quality were viewed on average significantly more often than higher quality videos. Patients and trainees may mistakenly assume that a greater number of video views indicates a more reliable or more educationally reputable video. Both patients and trainees alike should exercise caution when interpreting the findings of these videos and should certainly not use them as a sole means of education. The observation that YouTube videos may be misleading and present subpar educational material has been longstanding across a number of surgical and medical disciplines.^9^^,^^12^, ^13^, ^14^

It is unsurprising that the educational quality of these videos on YouTube is lacking given the lack of a true peer review process and how the algorithm may not necessarily prioritise videos of high educational quality. Easily accessible and high quality peer reviewed information for patient and trainee education is available through several hospital organisations and societies. For example, a brief scan of open access resources reveals that both the Canadian Society for Vascular Surgery and Society for Vascular Surgery and several hospitals publish high quality information for patient education.^47^, ^48^, ^49^, ^50^, ^51^ Similarly, the Society for Vascular Surgery has compiled what it deems to be reliable, educationally reputable resources online on https://svsondemand.vascular.org/.^52^ Notably, when these resources do include videos, they often link back to YouTube itself. The findings herein point to the need for a peer review process for these videos before they are published in the public domain and a need for strategies to identify high quality educational videos for patients and trainees.

YouTube and open access online education may play a particularly important role for trainees as it relates to procedures that are technically complex or those that trainees have decreasing or limited exposure to, such as carotid endarterectomy or open abdominal aortic aneurysm repair.^53^, ^54^, ^55^, ^56^ Trainees would probably benefit from simulation and pre-operative rehearsal in the form of watching these procedures before direct involvement in the operating room.^57^, ^58^, ^59^ In this study, the videos relating to trainee education for these pathologies were generally of poor to fair quality, and particular attention should be directed towards improving the quality of these educational videos as they may be of more utility to trainees.

The evaluation of YouTube as an educational platform has increased exponentially in the literature, with most of the studies in this paper being published in the last four years alone, a trend consistent with other specialties, with most studies being published in the last decade.^5^^,^^12^ The present study has identified several areas for improvement to augment the methodological rigor of this body of literature. Firstly, there is substantial heterogeneity in the scoring tools used to assess video quality, with 21 different scoring systems being used among the studies. Notably, 14 of these scoring systems were author generated and were often not externally validated. This presents challenges to the ability to compare educational quality across different studies. The use of standardised tools in future projects, such as the DISCERN score,^60^ JAMA score,^61^ and Global Quality Scale,^62^ is encouraged. Utility in author generated scales exists when it provides additional granularity into a disease state and when they are externally validated or adequately supported by contemporary evidence, such as the Abdominal Aortic Aneurysm Specific Score.^26^ Additionally, the areas that were most lacking according to the methodological quality assessment of individual studies according to the framework proposed by D'Souza et al. pertained to the reproducibility of the study, exploration of the limitations of the study, and provision of future directions. Future studies should consult these guidelines when planning studies that evaluate open access educational materials.^22^

Finally, it was found that many studies that evaluated YouTube as a source of patient or trainee education in vascular surgery either did not extract uploader source or were unable to identify an uploading source. This lack of source recognition is particularly worrisome in videos intended for trainees, where over 53% of videos had no identified uploader source. Lack of author identification may further contribute to confusion and difficulty with discerning video trustworthiness. The need for increased YouTube presence by academic and health institutions is encouraged, as has been suggested across the fields of urology, plastic surgery, and general medical education.^12^^,^^14^^,^^63^

### Strengths and limitations

This review has several limitations. Only videos shared to YouTube were included, excluding those from other video platforms or professional websites. This may create a bias towards content that is inherently unvetted and potentially of lower quality. YouTube specifically was chosen for this study due to its high traffic, popularity, and relatively global accessibility. Additionally, there was substantial heterogeneity in the educational quality assessment of individual studies, which precluded grouping together quantitative quality assessment ratings and instead having to rely on a global rating of poor, fair, or good. This reduces the precision of the estimate as it relates to the overall educational quality of videos. This is an intrinsic limitation of the study data set, and future reviews in this field may be able to perform a more precise analysis if there was more standardisation in the conduct and reporting of studies in this field, which may be made possible with the recommendations provided here. The highly sensitive search strategy is a strength of this review, increasing the number of studies for manual review in an effort to robustly review all available literature. Finally, while this paper suggests that the overall educational quality of vascular surgery YouTube videos is poor to fair, it is important to consider that there remain focal areas and channels with consistent high quality educational content which may be of particular benefit, such as the Houston Methodist DeBakey Cardiovascular Education videos.^64^

### Recommendations and next steps

This study demonstrates a need for higher quality YouTube videos in vascular surgery, such that they are created in correspondence with clinical guidelines and with reference to the peer reviewed literature. Academic incentives to publish on YouTube may be beneficial to improve overall video content, as suggested by Helming et al.,^12^ alongside institutions (academic journals, hospitals, university, etc.) working to produce high quality content. Additionally, a peer reviewed section on YouTube could perhaps be implemented to better support accurate information dissemination, as suggested by Farag et al.^65^ Increased awareness of open access websites, such as the SVS OnDemand: https://www.vasculartraining.org, will probably also be beneficial for the future education of patients and trainees. Future research efforts should aim to create a unified video quality assessment tool to reduce heterogeneity and allow for ease of comparison, Study authors should consult the guidelines by D'Souza et al. when conducting future work evaluating YouTube and other open access online materials.^22^ Finally, vascular surgery societies may consider creating checklists for the standardised production, distribution, and assessment of open access educational resources, including YouTube videos.

### Conclusion

YouTube is seeing increasing use by vascular surgery patients and trainees, and the literature in this field is seeing sustained growth. Despite this, the overall educational quality remains poor to fair, with most of the methodological rigor of individual studies being of fair quality. Moving forward, the standardised evaluation of educational videos on YouTube, the development of a peer review process and increased awareness of pre-existing high quality resources for patients and trainees are suggested. Additionally, the field would benefit from increased academic and healthcare institution presence on YouTube as it pertains to vascular surgery.

## Conflicts of interest

None.

## Funding

None.
