# Supplementary material for: YouTube as a Source of Patient and Trainee Education in Vascular Surgery: A Systematic Review
Source: EJVES Vasc Forum. 2024 Jan 26;61:62–76. doi: 10.1016/j.ejvsvf.2024.01.054 (PMC10897809; doi:10.1016/j.ejvsvf.2024.01.054)
Supplement: Multimedia component 1 [file mmc1.docx]

**Supplemental Table S1a.** MEDLINE search strategy and associated result count

Database(s): **Ovid MEDLINE: Epub Ahead of Print, In-Process & Other Non-Indexed Citations, Ovid MEDLINE® Daily and Ovid MEDLINE®**1946-Jan 19 2023
Search Strategy:

| **#** | **Searches** | **Results** |
| --- | --- | --- |
| 1 | Youtube.mp. | 3451 |
| 2 | Health*.mp. | 4503562 |
| 3 | Healthcare*.mp. | 349838 |
| 4 | information*.mp. | 1575824 |
| 5 | education*.mp. | 1151850 |
| 6 | surg*.mp. | 3569894 |
| 7 | or/2-6 | 9293611 |
| 8 | 1 and 7 | 2774 |

**Supplemental Table S1b.** Embase search strategy and associated result count check

Database(s): **Embase**1974 to Jan 19 2023
Search Strategy:

| **#** | **Searches** | **Results** |
| --- | --- | --- |
| 1 | Youtube.mp. | 4424 |
| 2 | Health*.mp. | 6273377 |
| 3 | Healthcare*.mp. | 562286 |
| 4 | information*.mp. | 2315357 |
| 5 | education*.mp. | 1496938 |
| 6 | surg*.mp. | 4774838 |
| 7 | or/2-6 | 12447811 |
| 8 | 1 and 7 | 3740 |

**Supplemental Table S1c.** Ovid Healthstar search strategy and associated result count

Database(s): **Ovid Healthstar**1966 to Jan 19 2023
Search Strategy:

| **#** | **Searches** | **Results** |
| --- | --- | --- |
| 1 | Youtube.mp. | 2074 |
| 2 | Health*.mp. | 3453690 |
| 3 | Healthcare*.mp. | 279646 |
| 4 | information*.mp. | 970619 |
| 5 | education*.mp. | 959624 |
| 6 | surg*.mp. | 2341784 |
| 7 | or/2-6 | 6452486 |
| 8 | 1 and 7 | 1759 |

| **Quality Assessment Instrument or Method** | **Number of uses, n** |
| --- | --- |
| Modified DISCERN /5 | 2 |
| JAMA Score | 2 |
| Global Quality Scale (GQS) | 2 |
| DISCERN | 1 |
| Medical information and content index (MICI) | 1 |
| HONcode score | 1 |
| Educational Assessment tool (EAT) | 1 |
| Author-created content assessment scale regarding lower limb arterial disease  by Dar et al. (2021) | 1 |
| Author-created usefulness scale regarding varicose veins  by Gunes et al. (2016) | 1 |
| Author-created abdominal aortic aneurysm specific score (AAASS)  by Radonjic et al. (2020) | 1 |
| Author-created score using guidelines to assess informational quality  regarding varicose veins by Kwok et al. (2017) | 1 |
| Author-created score on content, accountability, and production regarding endovascular aneurysm repair by Yan et al. (2021) | 1 |
| Author-created score on content regarding carotid endarterectomy  by Doenges et al. (2020) | 1 |
| Author created score on content and production regarding carotid endarterectomy  by Yan et al. (2020) | 1 |
| Author-created usefulness criteria regarding diabetic foot care  by Smith et al. (2019) | 1 |
| Author-created score on content regarding carotid endarterectomy  by Park et al. (2018) | 1 |
| Author-created score on content regarding inferior vena cava filters  by Kinariwala et al. (2018) | 1 |
| Author-created score on content regarding peripheral neuropathy  by Gupta et al. (2016) | 1 |
| Author-created usefulness criteria regarding diabetic foot care  by Abedin et al. (2015) | 1 |
| Author created score on content regarding peripheral neuropathy  by Gupta et al. (2015) | 1 |
| Author created score regarding diabetic foot care  by Dogan et al. | 1 |

**Supplemental Table S2: Quality Assessment Instruments and Methods Used Among the Studies**

| **Intended audience of videos** | **Patient** | **Trainee** | **Between Group Difference** |
| --- | --- | --- | --- |
| N, Studies | 18 | 6 | - |
| N, videos | 2999 | 222 | - |
| Studies rated as poor quality/studies that performed quality assessment of videos | 8/15 (53%) | 2/5 (40%) | P = 1.00^‡^ |
| Pooled mean video length [95% CI] | 5.9 [4.5, 7.4]  N studies = 11  N = 800  I^2^ = 92% | 9.7 [7.6, 11.8]  N studies = 5  N = 199  I^2^ = 0% | P < 0.01^†^ |
| Pooled mean views [95% CI] | 19030.7 [9663.9, 28397.5]  N studies = 12  N = 1285  I^2^ = 89% | 17582.0 [10605.2; 24558.7]  N studies = 4  N = 192  I^2^ = 48% | P = 0.81^†^ |
| Source of Upload | | | |
| Academic Affiliation/Institution | 5.5 [0.5, 10.5]  I^2^ = 88% | 6.7 [0.0, 16.5]  I^2^ = 86% | P = 0.83^†^ |
| Advertisers/Commercial/Media | 13.2 [3.2, 23.2]  I^2^ = 99% | 0.0 [0.0, 1.6]  I^2^ = 0% | P = 0.01^†^ |
| Clinic/Hospital/Healthcare Organization | 8.5 [0.0, 17.1]  I^2^ = 97% | 0.0 [0.0, 1.6]  I^2^ = 0% | P = 0.05^†^ |
| Patient/Public | 7.1 [3.0, 11.1]  I^2^ = 94% | 0.0 [0.0, 1.6]  I^2^ = 0% | P < 0.01^†^ |
| Physician | 10.6 [1.8, 19.3]  I^2^ = 97% | 24.2 [0.0, 54.9]  I^2^ = 98% | P = 0.40^†^ |
| Other HCP | 20.0 [10.6, 29.3]  I^2^ = 98% | 0.0 [0.0, 1.6]  I^2^ = 0% | P < 0.01^†^ |
| Other | 6.2 [1.2, 11.2]  I^2^ = 92% | 15.0 [0.0, 38.6]  I^2^ = 98% | P = 0.48^†^ |
| Not Reported | 25.1[5.3, 44.9]  I^2^ = 100% | 53.5 [15.3, 91.6]  I^2^ = 99% | P = 0.20^†^ |
| ^†^Subgroup interaction; ^‡^Fisher’s exact test. | | | |

**Supplemental Table S3: Study Characteristics Based on Target Audience**

| **Disease State** | **Academic affiliation/institution** | **Advertisers/Commercial/Media** | **Healthcare organization** | **Patient/Public** | **Physician** | **Other healthcare practitioner** | **Other** | **Not reported** |
| --- | --- | --- | --- | --- | --- | --- | --- | --- |
| Abdominal aortic aneurysms | 0.0 [0.0, 3.6]  I^2^ = 0% | 6.9 [0.0, 13.9]  I^2^ = 0% | 0.0 [0.0, 3.6]  I^2^ = 0% | 10.0 [0.0, 22.0]  I^2^ = 29% | 28.0 [0.0, 81.8]  I^2^ = 95% | 13.3 [0.0, 36.2]  I^2^ = 74% | 0.0 [0.0, 3.6]  I^2^ = 0% | 49.5 [0.0, 100.0]  I^2^ = 99% |
| Buerger's disease | 0.0 [0.0, 7.1]  I^2^ = N/A | 0.0 [0.0, 7.1]  I^2^ = N/A | 12.0 [4.5, 24.3]  I^2^ = N/A | 4.0 [0.5, 13.7]  I^2^ = N/A | 44.0 [30.0, 58.7]  I^2^ = N/A | 22.0 [11.5, 36.0]  I^2^ = N/A | 18.0 [8.6, 31.4]  I^2^ = N/A | 0.0 [0.0, 7.1]  I^2^ = N/A |
| Carotid artery stenosis | 8.7 [0.0, 26.1]  I^2^ = 94% | 0.0 [0.0, 1.7]  I^2^ = 0% | 0.0 [0.0, 1.7]  I^2^ = 0% | 0.0 [0.0, 1.7]  I^2^ = 0% | 28.1 [0.0, 83.3]  I^2^ = 99% | 0.0 [0.0, 1.7]  I^2^ = 0% | 29.4 [0.0, 73.0]  I^2^ = 99% | 33.2 [0.0, 98.5]  I^2^ = 100% |
| Deep vein thrombosis/Pulmonary embolism | 0.0 [0.0, 0.4]  I^2^ = 0% | 16.0 [0.0, 47.5]  I^2^ = 99% | 5.6 [0.0, 16.7]  I^2^ = 98% | 7.5 [0.0, 22.2]  I^2^ = 98% | 11.4 [0.0, 33.8]  I^2^ = 99% | 6.2 [0.0, 18.6]  I^2^ = 98% | 3.0 [0.0, 8.8]  I^2^ = 94% | 50.0 [0.0, 100.0]  I^2^ = 100% |
| Diabetic foot care | 12.3 [0.0, 26.9]  I^2^ = 93% | 8.4 [0.0, 18.8]  I^2^ = 94% | 0.1 [0.0, 0.7]  I^2^ = 0% | 11.1 [2.3, 19.9]  I^2^ = 96% | 11.1 [0.0, 26.2]  I^2^ = 98% | 27.0 [9.1, 44.9]  I^2^ = 99% | 12.1 [0.2, 24.0]  I^2^ = 96% | 14.3 [0.0, 42.3]  I^2^ = 100% |
| Lymphedema | 17.8 [10.5, 27.3]  I^2^ = N/A | 5.6 [1.8, 12.5]  I^2^ = N/A | 76.7 [66.6, 84.9]  I^2^ = N/A | 0.0 [0.0, 4.0]  I^2^ = N/A | 0.0 [0.0, 4.0]  I^2^ = N/A | 0.0 [0.0, 4.0]  I^2^ = N/A | 0.0 [0.0, 4.0]  I^2^ = N/A | 0.0 [0.0, 4.0]  I^2^ = N/A |
| Peripheral arterial disease | 0.0 [0.0, 1.9]  I^2^ = 0% | 0.0 [0.0, 1.9]  I^2^ = 0% | 0.0 [0.0, 1.9]  I^2^ = 0% | 0.0 [0.0, 1.9]  I^2^ = 0% | 19.5 [0.0, 58.4]  I^2^ = 96% | 15.5 [0.0, 46.2]  I^2^ = 97% | 0.0 [0.0, 1.9]  I^2^ = 0% | 64.5 [28.3, 100.0]  I^2^ = 97% |
| Varicose veins | 3.9 [1.4, 6.5]  I^2^ = 72% | 43.9 [5.1, 82.6]  I^2^ = 99% | 17.4 [0.0, 34.8]  I^2^ = 99% | 7.2 [1.4, 12.9]  I^2^ = 93% | 0.0 [0.0, 0.5]  I^2^ = 0% | 23.2 [2.2, 44.1]  I^2^ = 99% | 3.4 [0.0, 10.3]  I^2^ = 93% | 0.0 [0.0, 0.5]  I^2^ = 0% |
| Multiple | 4.4 [0.0, 18.1]  I^2^ = 58% | 0.0 [0.0, 0.3]  I^2^ = 0% | 0.0 [0.0, 0.3]  I^2^ = 0% | 0.0 [0.0, 0.3]  I^2^ = 0% | 0.0 [0.0, 0.3]  I^2^ = 0% | 0.0 [0.0, 0.3]  I^2^ = 0% | 0.0 [0.0, 0.3]  I^2^ = 0% | 95.6 [81.9, 100.0]  I^2^ = 58% |
| TOTAL | 5.7 [1.4, 10.0]  I^2^ = 84%  P < 0.01^†^ | 10.0 [2.2, 17.9]  I^2^ = 99%  P = 0.03^†^ | 6.5 [0.0, 13.0]  I^2^ = 96%  P < 0.01^†^ | 5.5 [2.2, 8.8]  I^2^ = 92%  P = 0.02^†^ | 14.0 [4.1, 23.9]  I^2^ = 97%  P < 0.01^†^ | 15.1 [7.3, 22.8]  I^2^ = 97%  P < 0.01^†^ | 8.6 [1.8, 15.4]  I^2^ = 95%  P = 0.02^†^ | 32.1 [14.1, 50.0]  I^2^ = 100%  P < 0.01^†^ |
| ^†^Subgroup interaction. | | | | | | | | |

**Supplemental Table S4**: Uploader Source and Video Topics Discussed

Note: since the above pooled proportions represent the true underlying proportion of population of YouTube videos, rather than the sample included in this study, and the weight instability of random-effect meta-analysis on multiple proportions, the sum of the estimates may not necessarily add up to 100%.

| **Author & Year** | **Sensible question** | **Comprehensive search** | **Reproducibility** | **Appropriateness of synthesis methods** | **Certainty of study findings** | | | | **Overall study quality** |
| --- | --- | --- | --- | --- | --- | --- | --- | --- | --- |
|  |  |  |  |  | **Limitations** | **Data adequacy** | **Inconsistency** | **Future direction** |  |
| Dar, 2021 | Y | Y | Y | Y | Y | Y | Y | N | Good |
| Wu, 2021 | Y | Y | Y | Y | N | Y | Y | N | Fair |
| Gunes, 2016 | Y | Y | Y | Y | N | Y | Y | N | Fair |
| Radonjic, 2020 | Y | Y | Y | Y | Y | Y | Y | N | Good |
| Kwok, 2017 | Y | Y | Y | Y | Y | Y | Y | Y | Good |
| Baytaroglu, 2021 | Y | Y | Y | Y | Y | Y | Y | Y | Good |
| Yan, 2021 | Y | Y | N | Y | N | Y | N | N | Fair |
| Doenges, 2020 | Y | Y | N | Y | N | N | Y | N | Fair |
| Yammine, 2020 | Y | Y | Y | Y | N | Y | Y | N | Fair |
| Yan, 2020 | Y | Y | N | Y | N | N | N | N | Fair |
| Cousins, 2020 | Y | N | N | Y | Y | N | Y | N | Fair |
| Smith, 2019 | Y | Y | Y | Y | N | Y | Y | Y | Good |
| Park, 2018 | Y | Y | N | Y | N | Y | Y | N | Fair |
| Kinariwala, 2018 | Y | N | N | Y | N | Y | N | N | Fair |
| Nominato, 2019 | Y | Y | Y | Y | N | Y | Y | Y | Fair |
| Bademci, 2017 | Y | Y | Y | Y | N | Y | Y | N | Fair |
| Pitcher, 2018 | Y | Y | N | Y | N | Y | Y | N | Fair |
| Gupta, 2016 | Y | Y | Y | Y | N | Y | Y | N | Fair |
| Abedin, 2015 | Y | Y | Y | Y | N | Y | Y | N | Fair |
| Gupta, 2015 | Y | Y | N | Y | N | Y | Y | N | Fair |
| Kucukakkas  2022 | Y | Y | Y | Y | Y | Y | Y | Y | Good |
| Dogan  2022 | Y | N | Y | Y | Y | Y | Y | Y | Good |
| Almaqhawi  2022 | Y | N | N | Y | Y | Y | Y | N | Fair |
| Celik  2022 | Y | Y | Y | Y | Y | Y | Y | Y | Good |
| **Total Percentage of Studies with Criterion Fulfilled** | 24/24 = 100% | 20/24 = 83% | 15/24 = 63% | 24/24 = 100% | 9/24 = 38% | 21/24 = 88% | 21/24 = 88% | 7/24 = 29% | Fair = 16/24 = 67%  Good = 8/24 = 33% |

**Supplemental Table S5: Study Quality Assessment**
